# Sam68 is a druggable vulnerability point in cancer stem cells

**DOI:** 10.1007/s10555-023-10145-8

**Published:** 2023-10-04

**Authors:** Amanda Mendes da Silva, Veronika Yevdokimova, Yannick D. Benoit

**Affiliations:** 1 Department of Cellular and Molecular Medicine, University of Ottawa, Ottawa, ON K1H 8M5 Canada; 2 School of Pharmaceutical Sciences, Faculty of Medicine, University of Ottawa, Ottawa, ON K1H 8M5 Canada

**Keywords:** Sam68, Cancer stem cell, SRC, CBP, Canonical wnt, Drug Discovery

## Abstract

Sam68 (Src associated in mitosis of 68 kDa) is an RNA-binding and multifunctional protein extensively characterized in numerous cellular functions, such as RNA processing, cell cycle regulation, kinase- and growth factor signaling. Recent investigations highlighted Sam68 as a primary target of a class of reverse-turn peptidomimetic drugs, initially developed as inhibitors of Wnt/β-catenin mediated transcription. Further investigations on such compounds revealed their capacity to selectively eliminate cancer stem cell (CSC) activity upon engaging Sam68. This work highlighted previously unappreciated roles for Sam68 in the maintenance of neoplastic self-renewal and tumor-initiating functions. Here, we discuss the implication of Sam68 in tumorigenesis, where central findings support its contribution to chromatin regulation processes essential to CSCs. We also review advances in CSC-targeting drug discovery aiming to modulate Sam68 cellular distribution and protein-protein interactions. Ultimately, Sam68 constitutes a vulnerability point of CSCs and an attractive therapeutic target to impede neoplastic stemness in human tumors.

## Introduction

Tumor functional heterogeneity has been extensively characterized in cellular constituents of leukemias and solid tumors [1, 2]. Numerous cellular mechanisms have been associated with such heterogeneity, including genetic and epigenetic alterations, as well as interactions with the tumor microenvironment [3–5]. Cancer Stem Cells (CSCs) represent a rare cellular constituent of tumor heterogeneity sustaining tumor growth and metastasis via hallmark functions like self-renewal and cancer-initiating capacities [6–8]. Lineage-tracing experiments and in vivo repopulation assays revealed the dynamic nature of CSCs within the tumor mass via cellular plasticity [9, 10]. Experiments showing the reestablishment of CSC pools following targeted ablation of self-renewing cells within tumors confirmed that cancer stemness consists of a cell state rather than a definite cell type [9, 11]. Thus, the “CSC state” is closely related to quiescence or “dormancy” within tumor heterogeneity, which reduces susceptibility to anti-mitotic and radiation-based therapies [12]. Early dissemination of dormant CSC-like cells can cause relapse and growth of metastasis several years after the elimination of the initial primary tumor [12, 13]. The maintenance of CSC functions and associated therapeutic resistance mechanisms is mediated by profound alterations in the chromatin organization and transcriptional regulatory networks [5]. Considering CSCs as the root of cancer, the identification of druggable pathways that are essential to neoplastic stemness, but of minor importance to maintain healthy tissue integrity, constitutes the basis for the development of improved anticancer therapeutic tools.

**Fig. 1:**
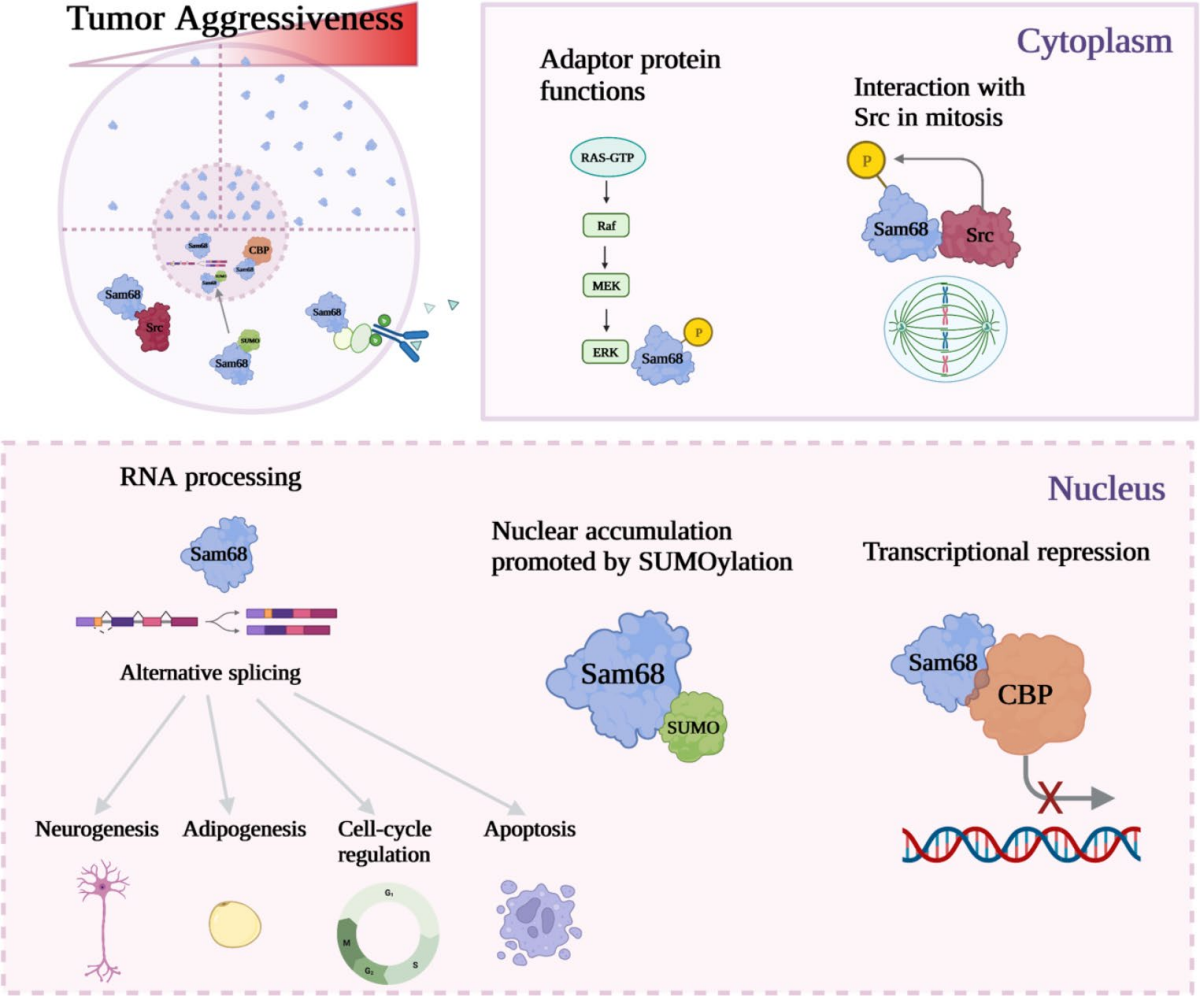
Subcellular distribution of Sam68 vs. tumor aggressiveness and overview of localization-specific functions of Sam68

Among the current pharmacological strategies effective at targeting CSC reservoirs in pre-clinical settings, many were centered around pro-oncogenic chromatin regulation pathways, including DNA methylation machinery and histone-modifying complexes [14]. For instance, the inhibition of the histone methyltransferase G9a is gaining importance as a promising therapeutic target to block cancer stemness in preclinical studies [15–17]. Moreover, the inhibition of histone acetyltransferases (HATs) was shown to impair CSC functions in different contexts. Of note, a comprehensive study by MacPherson et al. identified the MYST domain-containing acetyltransferase HBO1 as an essential contributor to H3 lysine-14 acetylation (H3K14ac) in myeloid leukemia stem cells, facilitating the transcription of stemness genes [18]. CREB-binding protein (or CBP) is another HAT extensively linked to cancer stemness, as a co-factor for the transactivation of Wnt/ β-catenin target genes [19, 20]. The recruitment of CBP at chromatin-bound TCF/β-catenin complex maintains gene expression programs promoting self-renewal, enhanced survival, and sustained undifferentiated state via acetylation of H3K14 and H3K18 [20]. Antagonizing the co-factor functions of CBP with the reverse-turn peptidomimetic small molecule ICG-001 (Table 1) selectively induced apoptosis in colon cancer cells and reduced the formation of intestinal adenomas in vivo [21]. While the peptidomimetics ICG-001 and its analog CWP232228 (Table 1) were initially thought to inhibit Wnt/β‑catenin signaling by disrupting the interactions between β-catenin and the histone acetyltransferase CBP [21–23], two studies highlighted the essential partaking of Sam68 in such molecular mechanism of action leading to CSC-specific repression of canonical Wnt targets [20, 24]. As extensively discussed below, reverse-turn peptidomimetics promote nuclear accumulation of Sam68 and the sequestration of CBP from chromatin-bound TCF/β‑catenin complexes in CSC models [20, 24]. To contextualize, high cytoplasmic levels of Sam68 were associated with more aggressive tumors and enhanced pro-oncogenic functions compared to the cases showing prominent nuclear localization (Fig. 1) [25–27].

**Table 1:** Small molecules currently described as pharmacological modulators of Sam68 functions

Compounds	Experimental parameters (IC_50_ or EC_50_)
**UCS15A** [58, 117]	3 µM (inhibit bone resorption in MNC cells) 100–200 µM (disruption of SH3 mediated protein-protein interaction in HCT116) 6.6 µM (inhibit bone resorption in ex vivo) 1 µM (HCT116 and analysed by IP)
**ICG-001** [20, 24]	3 µM (extrapolated on to the breast cancer cell lines, MDA-MB231 and MCF7 and colon cancer cell lines HT29 and SW480) 3 µM in t-hESCs > 20 µM in HIEC (normal) 14.6 µM in HT29
**CWP232228** [20, 24]	0.5 µM (extrapolated on to the breast cancer cell lines, MDA-MB231 and MCF7 and colon cancer cell lines HT29 and SW480) 0.1 µM t-hESCs > 20 µM in HIEC (normal) 1.47 µM in HT29 0.11 µM in t-hESC 100 mg/kg in Murine syngeneic serial tumor transplantation model
**YB-0158** [24]	0.01 µM in t-hESC 4.40 µM in HIEC (normal) 0.28 µM in HT29 0.37 µM in HCT116 3.25 µM in SW480 0.125 to 2 µM in patient-derived colorectal tumor organoids 100 mg/kg in Murine syngeneic serial tumor transplantation model

Notably, the effect of ICG-001-like peptidomimetics on Sam68 nuclear accumulation was not observed in healthy human stem cells, and no impairments of normal hematopoiesis or intestinal tissue architecture were detected upon in vivo treatments [20, 24]. Thus, indications that Sam68 may serve as a context-specific handle to curb hyperactive canonical Wnt/β-catenin activity and other CSC-supporting transcriptional networks, without disrupting healthy tissue integrity, is of particular interest for the development of next-generation therapeutics targeting cancer at its roots [24]. Here, we review the main functions of Sam68 and their implication in tumorigenesis and the regulation of chromatin dynamics. We also explore future avenues for therapeutics development hindering Sam68 in CSC populations.

## Sam68 is a multifunctional protein involved in homeostasis

Sam68 (Src-Associated in Mitosis of 68 kDa), also known as KH domain containing RNA binding signal transduction associated protein 1 (KHDRBS1), belongs to the STAR (Signal Transducer and Activator of RNA) family of RNA-binding proteins [28, 29] and participates in numerous cellular functions, including RNA processing [30, 31], transcription [32, 33], kinase- and growth-factor-signaling [34, 35], cell-cycle regulation, and apoptosis [36, 37] (Fig. 1). Sam68 is also involved in responses to external factors such as hepatic gluconeogenesis [38] and pro-inflammatory pathways [39, 40]. Furthermore, Sam68 has been documented to mediate cell fate commitment in numerous systems, including germ cells [41, 42], neurogenesis [43, 44], and adipogenesis [44, 45] via its regulatory role on alternative splicing (Fig. 1).

### Sam68 in alternative splicing

Sam68 has been related to numerous aspects of RNA metabolism including modifying mRNA stability and/or mRNA translation [46], alternative splicing [47–50], and polysomal recruitment of mRNA [51–53]. Alternative splicing is a critical posttranslational process where the exon portions of a specific mRNA will be aligned in different ways to produce distinct mature mRNAs. Thus, alternative splicing contributes to protein diversity by enabling each tissue or cell type to generate multiple isoforms out of a single pre-mRNA. The first mechanism of alternative splicing regulation by Sam68 in response to extracellular was described by Matter et al., where ERK-dependent phosphorylation of Sam68 promoted the inclusion of v5-exon in CD44 [47]. The role of Sam68 in CD44-v5 splicing was later shown to facilitate EGF-dependent cell migration [34].

The role of Sam68 in alternative splicing was extensively linked to neurogenesis and spermatogenesis. In a study by Iijima and colleagues, Sam68 knockout mouse brains revealed severe perturbations of neurexin-1 (Nrxn1) splice variants, establishing Sam68 as a key regulator of site-specific and activity-dependent splicing of Nrxn1 within cerebellar neurons [31]. Moreover, Sam68 was shown to induce exon 7 skipping in the Survival of Motor Neuron 2 (SMN2) transcript, preventing the rescue of mutated SMN1 in spinal muscular atrophy pathogenesis [54]. Sam68 also regulates alternative splicing of epsilon sarcoglycan (Sgce) by repressing the inclusion of exon 8, which fosters neuronal differentiation in mouse pluripotent cells [43]. In spermatogenesis, skipping of exon 8 in Sgce depends on Sam68 expression and nuclear localization, where it interferes with the recruitment of the general splicing factor U2AF65 [42]. In the broader context of spermatogenesis, Sam68 nuclear localization was found to rely on the integrity of cellular RNA and tightly associated to alternative splicing regulation within transcriptionally active regions during germ cell differentiation [42].

Additionally, Sam68 regulates the alternative splicing of both mTOR and Ribosomal S6 Kinase B1 (RPS6KB1) to promote adipogenesis [55, 56]. Importantly, Paronetto et al. showed that Sam68 can regulate apoptosis via alternative splicing of the Bcl-x transcript, where fluctuations in Sam68 subnuclear distribution affect the ratio of antiapoptotic Bcl-x(L) versus proapoptotic Bcl-x(s) mRNA [48]. In this process, the affinity of Sam68 for Bcl-x mRNA depends on its phosphorylation by the Src-like kinase Fyn [48].

In cancer, Sam68 was shown to promote a prooncogenic energy metabolism switch in lung adenocarcinoma by influencing alternative splicing of Pyruvate Kinase Muscle (PKM) [50]. Specifically, the interaction of Sam68 with the RGG motif of hnRNP A1 was shown to favor the exclusion of exon-9 at the expense of exon-10 through PKM pre-mRNA processing, yielding increased levels of the PKM2 isoenzyme. PKM2 confers cancer cells with a metabolic advantage over PKM1 expression by enhancing aerobic glycolysis [50]. Other studies also related Sam68 to PKM alternative splicing and PKM2 overexpression in colorectal cancer [49].

### Sam68 as an adaptor protein

Upon its first description, Sam68 was reported to be physically associated with activated Src protein during fibroblast mitosis, leading to its phosphorylation on tyrosine residues (Fig. 1) [28, 57]. Such an interaction was shown to occur through Sam68 proline-rich motifs binding to Src SH3 domains [28, 57, 58]. The phosphorylation of C-terminal tyrosine and threonine residues was also shown to foster the participation of Sam68 to multiple signal transduction pathways (Fig. 1), including T cell receptors, MAPK, PI3K/AKT, and JAK/STAT cascades, as an adaptor protein [27, 59]. Such phosphorylation-dependent functions of Sam68 were suggested to be mutually exclusive to its RNA-binding functions [27].

For instance, Sam68 binds to the SH3 domains in a multitude of non-RNA partners, including PI3K [60, 61], phospholipase C gamma-1 (PLCγ-1) [62], Ras-GAP [63], and MAPKs. These latter were shown to phosphorylate Sam68 via ERK to facilitate CD44v5 splicing and HGF-dependent cell migration [64, 65]. Sam68 also exhibits scaffolding functions in response to environmental stimuli, such as EGF [66], HGF [67], and TNF-alpha [68] upon binding to their respective receptor tyrosine kinases. Multiple studies highlighted the implication of Sam68 in mediating TNF-α/NF-kB axis activation to sustain pro-survival signals in different contexts [32, 35, 38]. Most of the Sam68 interactions as a docking protein were comprehensively reviewed elsewhere [69, 70].

## Sam68 in cancer

The upregulation of Sam68 expression and its subsequent impact on proliferation and tumorigenicity has been reported in several cancers, such as breast [71, 72], prostate [73], cervical [25], gastric [74], non-small cell lung [75] and renal tumors [26]. Although Sam68 manifests predominantly nuclear localization, driven by a nuclear localization signal (NLS) in the C-terminus [76], previous reports have shown the relationship between Sam68 localization and its different cellular functions (Fig. 1). Post-translational modifications including threonine and tyrosine phosphorylation, arginine methylation, and SUMOylation affect Sam68 cellular localization, its RNA binding affinity, and modulate its interaction with different signaling proteins (Fig. 1) [29].

While Sam68 is mainly present in the nucleus of most cell types, Huot et al. reported increased cytoplasmic Sam68 levels in freshly adhering cells. In such conditions, cytoplasmic Sam68 was observed close to the plasma membrane, where it acts as an adaptor to modulate Src activity [34]. Consistently, phosphorylation of T33 and T317 by Cdk1 was shown to positively influence Sam68 cytoplasmic distribution and pro-oncogenic functions in colorectal cancer cells. Ectopic expression of a constitutive phospho-mimetic mutant of Sam68 (T33E/T317E) in HCT116 cells demonstrated reduced RNA binding capacities concomitant with higher cytoplasmic localization [27]. Moreover, Cdk1-driven Sam68 cytoplasmic localization decreased apoptosis susceptibility and enhanced proliferation of HCT116 colorectal cancer cells [27]. Interestingly, Zhang et al. have established a relationship between Sam68 subcellular localization and renal cancer prognosis, where high expressions of Sam68 and greater cytoplasmic distribution were both predictive of poor survival outcomes (Fig. 1) [26]. Cytoplasmic distribution of Sam68 was also more frequently observed in advanced clinicopathological stages of the disease (III-IV: 48.1%) vs. earlier stages (I-II: 31.6%) [26]. In an independent study, Li et al. showed that elevated Sam68 expression and cytoplasmic localization were also significantly associated with poor prognosis in cervical cancer, including a higher prevalence of pelvic lymph node metastasis, as well as shorter disease-free and overall patient survival [25].

### Cytoplasmic Sam68 and TNF-dependent signaling

Cytoplasmic Sam68 was found essential to TNFR-dependent signaling in HeLa cells, where it forms two distinct complexes with RIP1, respectively mediating pro-survival NF-kB activation (Complex-I), and TNF-induced extrinsic apoptosis (Complex-II) [35]. In this context, Sam68 silencing did not interfere with the basal expression of most NF-kB target genes in the absence of TNF-alpha stimulation [35]. Similarly, the recruitment of Sam68 to membrane-bound FADD/Caspase-8 complexes is only essential to TNF-induced apoptosis and was dispensable for Fas-dependent Caspase-8 activation [35]. While these observations were not directly studied in the tumorigenic framework of HeLa cells, they underline the complexity of Sam68-dependent regulation of cell survival in cancer.

### Sam68 is facilitating the nuclear translocation of oncogenic Vav1

Vav1 is an SH3 domain-containing GDP/GTP nucleotide exchange factor (GEF) which can be mutated in different types of tumors to actively support tumorigenesis [77]. While typically present in the cytoplasm where it regulates the actin cytoskeleton, Vav1 can interact with Sam68 via its C-terminal SH3 domain (Vav1SH3C) and cause nuclear accumulation of Vav1 [78]. The D797N oncogenic mutations in Vav1SH3C, which have been found in human cancers, maintain their capacity to bind Sam68 [78, 79]. Co-transfection experiments revealed that Sam68 overexpression with oncogenic D797N Vav1 (oncVav1) leads to the transformation of NIH3T3 fibroblasts, while transfection of Sam68 only has no immediate impact on this process [78]. Altogether, these observations suggest a potential pathway through which cytoplasmic Sam68 could influence tumor-initiating events and cancer progression (Fig. 1).

### Sam68 contributes to nuclear signal transduction in cancer

The role of Sam68 as a constituent of key signaling pathways is not limited to the cytoplasm and was documented at different levels in the nucleus of cancer cells. For instance, Sam68 expression was shown essential to robust induction of histone variant H2AX phosphorylation (gH2AX) and efficient repair of DNA double-strand breaks (DSBs) in human osteosarcoma cells upon g-irradiation [80]. In Sam68-deficient conditions, the phosphorylation of ATM, one of the kinases phosphorylating H2AX, as well as its substrates Chk1 and Chk2, is lower than in Sam68-sufficient osteosarcoma cells in response to g-irradiation-induced DSBs. Mechanistic studies demonstrated that Sam68 interacts with the enzyme poly(ADP-ribose) polymerase 1 (PARP1), which enables the synthesis of polymers of ADP-ribose (PAR) in response to radiation-induced DSBs. In turn, PARs are required to phosphorylate ATM, Chk1, Chk2, and H2AX, participating in the DSB repairing process. Similarly, the Sam68-dependent induction of PAR synthesis was shown to be critical for the transactivation of anti-apoptotic NF-kB target genes in colorectal cancer cells subjected to genotoxic stress [81]. This phenomenon was further investigated in vivo, where Sam68 knockout mice displayed increased radiosensitivity due to impeded PAR synthesis and NF-kB activation [82]. Overall, these findings underscore the importance of Sam68 as a nuclear signal transducer conferring cancer cells with enhanced resistance capacities versus genotoxic stress.

### Nuclear Sam68 acts as a transcriptional co-regulator in cancer

Despite the association between cytoplasmic distribution and poor prognosis in cancer patients, several pro-oncogenic functions of Sam68 were described in the nucleus. Accordingly, nuclear Sam68 was reported to enhance the recruitment of activated NF-kB complex to the promoter of intercellular adhesion molecule-1 (ICAM-1) in the context of fatty acid deficiency in ovarian cancer [83]. Sam68-dependent induction of ICAM-1 in lipophagic cells was associated with tumor progression and contributed to poor survival in patients with ovarian carcinoma [83]. Similarly, in Epstein-Barr virus (EBV) associated gastric carcinoma, Sam68 acts as a transcriptional co-activator regulating the expression of the pro-oncogenic m^6^A methyltransferase METTL3 [84]. In this context, Sam68 interacts with the EBV circular RNA circRPMS1 and facilitates its recruitment to the METTL3 promoter [84]. Increased METTL3 expression due to Sam68 co-activator functions results in enhanced proliferation, migration, invasion, and anti-apoptosis signaling in primary gastric tumors and distant metastasis [84].

In contrast, Sam68 was reported as a co-repressor of cyclin D1 expression in Ewing sarcomas, by promoting the formation of a multimolecular complex with the RNA/DNA helicase DHX9 and the promoter-associated non-coding RNA pncCCND1_B [85]. Stimulation of the insulin growth factor (IGF) mitogenic pathway or the presence of the oncogenic EWS-FLI1 fusion protein were shown to disrupt Sam68/DHX9 interactions, leading to the upregulation of cyclin D1 expression. Therapeutic strategies maintaining the integrity of the Sam68/DHX9/pncCCND1_B complex were proposed against Ewing sarcomas [85]. Similarly, Sam68 was reported as a transcriptional repressor by physically interacting with the multifunctional transcriptional co-factor CBP in leukemia and breast cancer cells (Fig. 1) [86]. Hong et al. established that Sam68 binds to the CBP CH3/TAZ2 domain via a conserved FXE/DXXXL motif [86]. Such an association with CBP in the nucleus of cancer cells is independent of Sam68 RNA-binding activity and interferes with general CBP transcriptional co-activator functions (Fig. 1), as demonstrated by GAL4-CBP fusion reporter assays in U2OS cells [86]. The upregulation of nuclear Sam68 in different cancer models, including leukemia and colorectal cancer, also decreased CBP recruitment at Wnt/β‑catenin target promoters and was associated with transcriptional repression [20, 24]. This phenomenon was explained by a shift in CBP binding partner interaction, from β‑catenin to Sam68, following cytoplasmic-to-nuclear translocation of Sam68 and was linked to reduced tumorigenic activities [20, 86].

In colorectal cancer cells, nuclear Sam68 was also described as a transcriptional co-activator of p53, where Sam68 and p53 interact in an RNA-dependent manner and form a scaffold for the recruitment of additional co-activators such as CBP or PRMT1 [87]. In this context, Sam68 constitutes an essential component of the p53 pathway regulating tumor suppression.

These contrasting examples of nuclear Sam68 influencing gene expression in cancer suggest context-specific roles for this protein, where molecular switches are required to determine whether Sam68 acts as a pro or anti-tumorigenic factor.

### Nuclear Sam68 and alternative splicing in cancer

The ability of Sam68 to regulate alternative splicing of certain transcripts has also been documented in the cancer (Fig. 1). Specifically, Sam68-dependent alternative splicing of Cyclin D1 (CCND1) pre-mRNA results in the accumulation of the Cyclin D1b transcript, which displays a stronger oncogenic potential [88, 89]. Moreover, this process is regulated by ERK1/2 and Src kinases, which oppositely influence the affinity of Sam68 for the target RNA, and consequently, the Cyclin D1b/Cyclin D1a ratios [88]. Sam68 also contributes to epithelial-to-mesenchymal transition (EMT) in colorectal cancer models, where ERK-dependent phosphorylation of Sam68 upregulates the levels of proto-oncogene SF2/ASF transcript [90]. This process was shown to rely on the implication of Sam68 in the alternative splicing nonsense-mediated mRNA decay (AS-NMD) pathway [90]. Consistently, downregulating Sam68 expression in cervical cancer reversed EMT by inhibiting the Akt/GSK-3β/Snail pathway [25]. While this impact of Sam68 silencing was not directly related to alternative splicing, it further supports cellular plasticity (represented by EMT) as another Sam68-dependent mechanism influencing tumorigenesis.

### Spatial distribution of nuclear Sam68 in cancer

Research on the spatial distribution of Sam68 has revealed its presence in specialized multiproteic structures known as Sam68 nuclear bodies (SNBs) appearing as punctated foci in the nucleus by immunofluorescence [91]. Such SNBs are membrane-less organelles originating from liquid-liquid phase transitions, ranging from 0.3 to 1 μm in size, trafficking RNAs through the nucleoplasm [91, 92]. These dynamic structures contain different Sam68-associated proteins, including heterogeneous nuclear ribonucleoproteins (hnRNPs), Sam68-like mammalian proteins SLM-1/2, and the splicing-associated factor YT521, and are predominantly localized in peri-nucleolar areas [91–94]. SNBs were observed at high levels in different types of neoplasms, such as cervical, breast, and bone tumor cells, whereas they are less common (< 1%) in normal cells (e.g., fibroblasts) [91, 94]. Furthermore, other cell lines such as MCF-7 and Neuro-2 A exhibit lower prevalence of SNBs, suggesting variations among cell types or degree of malignancy [91]. The dysregulation of protein condensates defined by liquid-liquid phase separation can impact various cellular processes, such as intracellular signaling, chromatin organization, and transcriptional regulation [95]. Thus, the SNB abundance and distribution on cancer cells could have a profound implication on fundamental processes such as transcriptional, epigenetic, and metabolic reprogramming [95].

Taken together, the role of Sam68 in tumorigenesis appears highly context-specific, where it depends on tumor types, subcellular distribution, environmental factors, and the implication of its RNA binding activity versus adaptor protein functions (Fig. 1). Thus, further investigation will be essential to decipher the exact contributions of Sam68 in distinct oncogenic conditions.

## Sam68 represents a vulnerability point in cancer stem cells

Studies have demonstrated the involvement of Sam68 in the promotion of stem-associated functions in different types of systems, including neural progenitors and breast tumors [96, 97].While Sam68 was found overexpressed in several cancers, its distribution is not uniform across tumor cellular heterogeneity, where Sam68 levels substantially differ among tumor functional compartments. Specifically, Sam68 protein levels are significantly elevated in breast (CD24^lo^/CD44^hi^, MDA-MB-231) and colorectal (CD133^hi^/CD24^hi^/CD44^hi^, HT29) CSC-like populations, based on characteristic surface marker expression profiles, versus respective non-tumorigenic cell counterparts [20]. In breast cancer, Myc-overexpressing mammospheres enriched with cells presenting enhanced DNA repair capacities and in vivo CSC functions presented significantly higher Sam68 expression compared to healthy mammary progenitors [72]. In this context, elevated Sam68 expression hindered the efficacy of therapeutics targeting cycline-dependent kinases (dinaciclib) and DNA repair machinery (B02, Rad51 inhibitor). Interestingly, Sam68 deletion showed synthetic lethality when combined with Rad51 inhibition in breast tumor serial transplantation assays [72].

In colorectal cancer, the deletion of Sam68 significantly reduced tumor growth in a murine genetic model of spontaneous tumorigenesis, without notable impact in the healthy colonic mucosa [98]. Similarly, Sam68 is overexpressed in human primary samples nonmelanoma skin cancer (NMSC) compared to healthy skin tissues, and its silencing impairs anchorage-independent colony formation capacities in NMSC cells [99]. In a murine genetic model of NMSC, Sam68 deletion significantly delayed the onset of tumorigenesis [99]. Taken together, these observations suggest a key role for Sam68 in the maintenance of CSC-associated functions and support its unique aspect as a vulnerability point to impede self-renewal and cancer-initiating capacities.

### Sam68 promotes self-renewal in normal and tumor tissues

Gene deletion experiments identified Sam68 as essential to maintain the pool of neural progenitor cells (NPCs) in mouse cortex development, where Sam68 knockout embryos exhibited accelerated NPC differentiation into post-mitotic neurons [97]. Sam68-deficient NPCs isolated from E13.5 mouse cortices showed impaired clonogenicity and increased differentiation compared to wild-type counterparts, associating elevated Sam68 expression to self-renewal capacity [97]. Such a positive impact of Sam68 on self-renewal was linked to its implication in alternative splicing regulation. Specifically, high levels of Sam68 prevented splicing events leading to premature termination in the transcript of Aldh1a3, which promotes glycolytic metabolism and self-renewal in NPCs [97]. Aldh1a3 itself sustains essential metabolic aspects of neoplastic stemness and its expression was associated with self-renewal in glioma stem cells [100, 101]. Therefore, Sam68 is proposed to mediate the balance between self-renewal and differentiation cells in the developing cortex, which suggests a similar role for Sam68 in different tissues and pathophysiological contexts. Accordingly, Sam68 was enriched in CSC-like side populations sorted from human breast cancer models, while knockdowns reduced mammosphere formation frequency [96]. Overall, this strengthens the concept of Sam68 as a critical mediator of self-renewal in cancer.

### Sam68 in the regulation of the WNT/β-catenin pathway

**Fig. 2:**
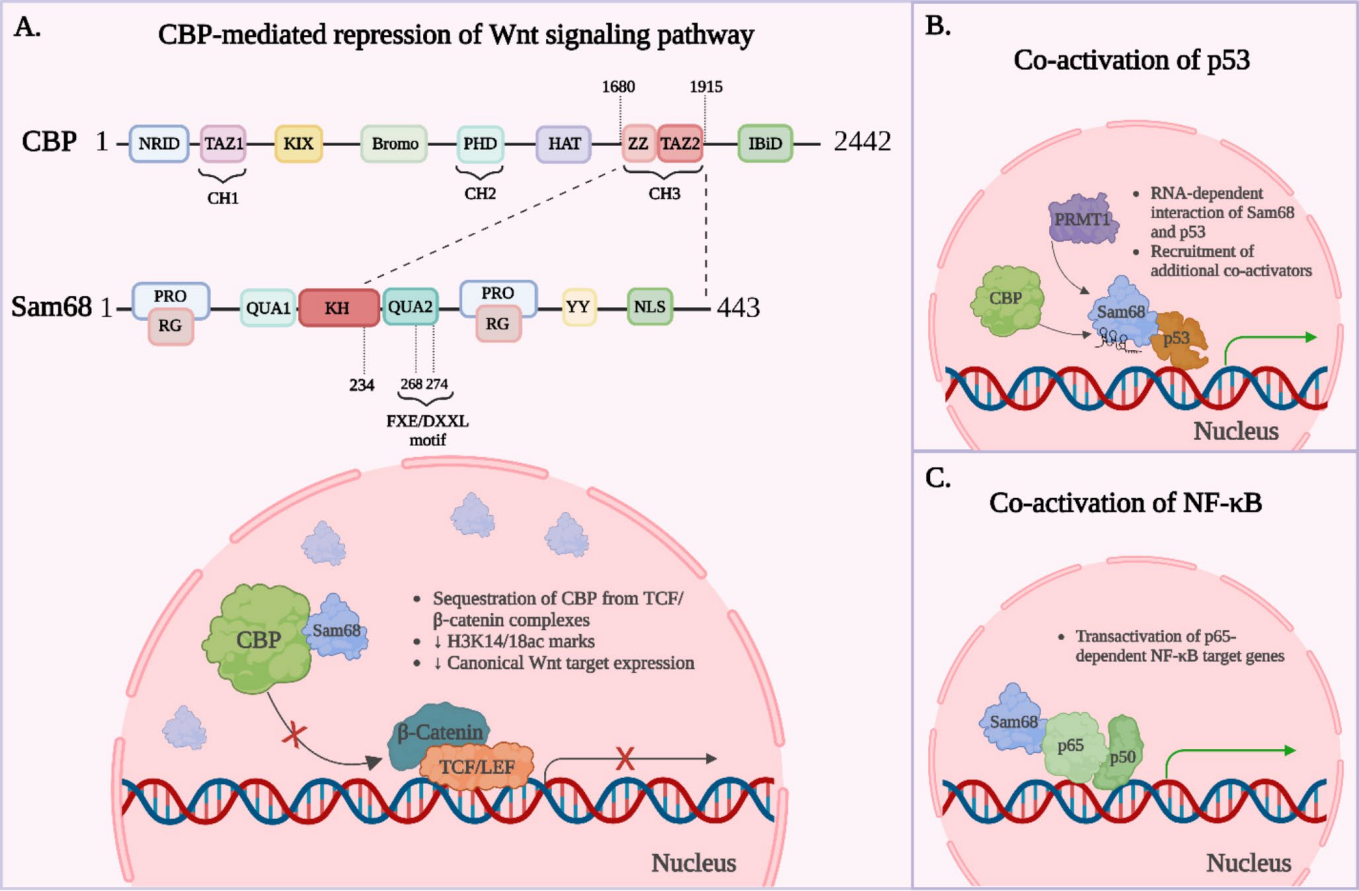
Functions of Sam68 as an epigenetic modulator of signaling in CSC. **(A)** Representation of the interaction of Sam68 with CBP and a mechanism of subsequent repression of Wnt signaling pathway. **(B)** Representation of RNA-dependent co-activation of p53 signaling by Sam68. **(C)** Schematic representation of Sam68-mediated transactivation of NF-kB signaling

Besides its influence on glycolytic metabolism, Sam68 was also tied to self-renewal by regulating the canonical Wnt cascade, which plays a pivotal role in stemness and cell fate throughout development and in adult tissues [102, 103]. This pathway is frequently hyperactivated in cancer, playing an important role in maintaining self-renewal in CSCs [104]. Recent studies manipulating Sam68 subcellular distribution observed that canonical Wnt target genes were downregulated when Sam68 was targeted to the nucleus in colorectal cancer cells and transformed human embryonic stem cells (t-hESCs) [20, 24]. Inside the nucleus of cancer cells, Sam68 can associate with the HAT and canonical Wnt co-activator CBP to exert a transcriptional repressive role, which is independent of its RNA binding functions (Fig. 2) [86].

Moreover, Pygopus 2 (Pygo2) is another canonical Wnt co-activator, which binds to trimethylated H3K4 loci to promote cancer progression and dedifferentiation [105]. Pygo2 nuclear localization and its impact on Wnt target genes depend on interactions with β‑catenin bound CBP, where Pygo2 gets transiently acetylated [106]. Specifically, acetylation of Pygo2 by CBP promotes its nuclear localization, while p300-dependent acetylation is translocating Pygo2 to the cytoplasm. Considering that Pygo2 is acting on Wnt targets while being present in the nucleus, such a dichotomy between Pygo2 responses to acetylation may explain why CBP-Wnt signaling promotes cell proliferation, while p300-Wnt signaling is associated with differentiation [106]. Recently, Sam68 nuclear accumulation was suggested as a repressor of Pygo2-dependent canonical Wnt activation in colorectal cancer, where enhanced formation of CBP-Sam68 complexes would favor Pygo2 acetylation by p300 and subsequent nuclear export [107]. This interplay between Sam68 and Pygo2 is susceptible to control the pro-proliferation versus pro-differentiation/apoptosis activation balance of the canonical Wnt pathway according to the “just-right model” in colorectal cancer [107, 108]. This concept presents Sam68 as a critical mediator of the Wnt/β-catenin activation state in cancer through its association with CBP, which could be harnessed in the development of selective therapeutic strategies hampering neoplastic self-renewal.

### Sam68 as a selective switch between major pathways in CSCs

In addition to canonical Wnt/β-catenin targets, Sam68 was shown to regulate the expression of genes under the control of transcription factors such as p53 and NF-kB in cancer (Fig. 2) [87, 98]. While Sam68 is essential to transactivate anti-apoptotic NF-kB target genes, it also acts as a selective co-activator of stress-activated p53 to upregulate cell cycle checkpoint genes in colorectal cancer cells [87]. Such observations highlight the fundamental role of Sam68 in selectively orchestrating transcriptional regulators across specific functional gene networks in cancer. It has been proposed that post-translational modifications of Sam68 might dictate its transcriptional specificity, where SUMOylation of Sam68 would enhance interactions with CBP and resulting Wnt/β-catenin inhibition, whereas S113/117 phosphorylation of Sam68 would promote the transactivation of NF-kB target genes [109]. Recently, the O-GlcNAcylation on N-terminal serine residues of Sam68 was associated with poor survival outcomes and later disease stages in human lung adenocarcinomas. Generally, O-GlcNAcylated Sam68 was reported to promote migration and invasiveness, although no clear molecular mechanism was described [110]. Further investigation may uncover distinct subcellular localization patterns for Sam68 marked with this post-translational modification and specialized transcriptional functions influencing CSC biology.

Pro-oncogenic alteration of Wnt/β-catenin, NF-kB, and p53 pathways and their respective target genes were extensively associated with CSCs in different types of tumors [104, 111, 112]. Thus, it is reasonable to consider Sam68 as a central regulator of larger genetic networks sustaining CSCs according to various environmental stimuli (Fig. 2).

## Pharmacological targeting of Sam68 in cancer stem cells

### The reverse-turn peptidomimetic avenue

**Fig. 3:**
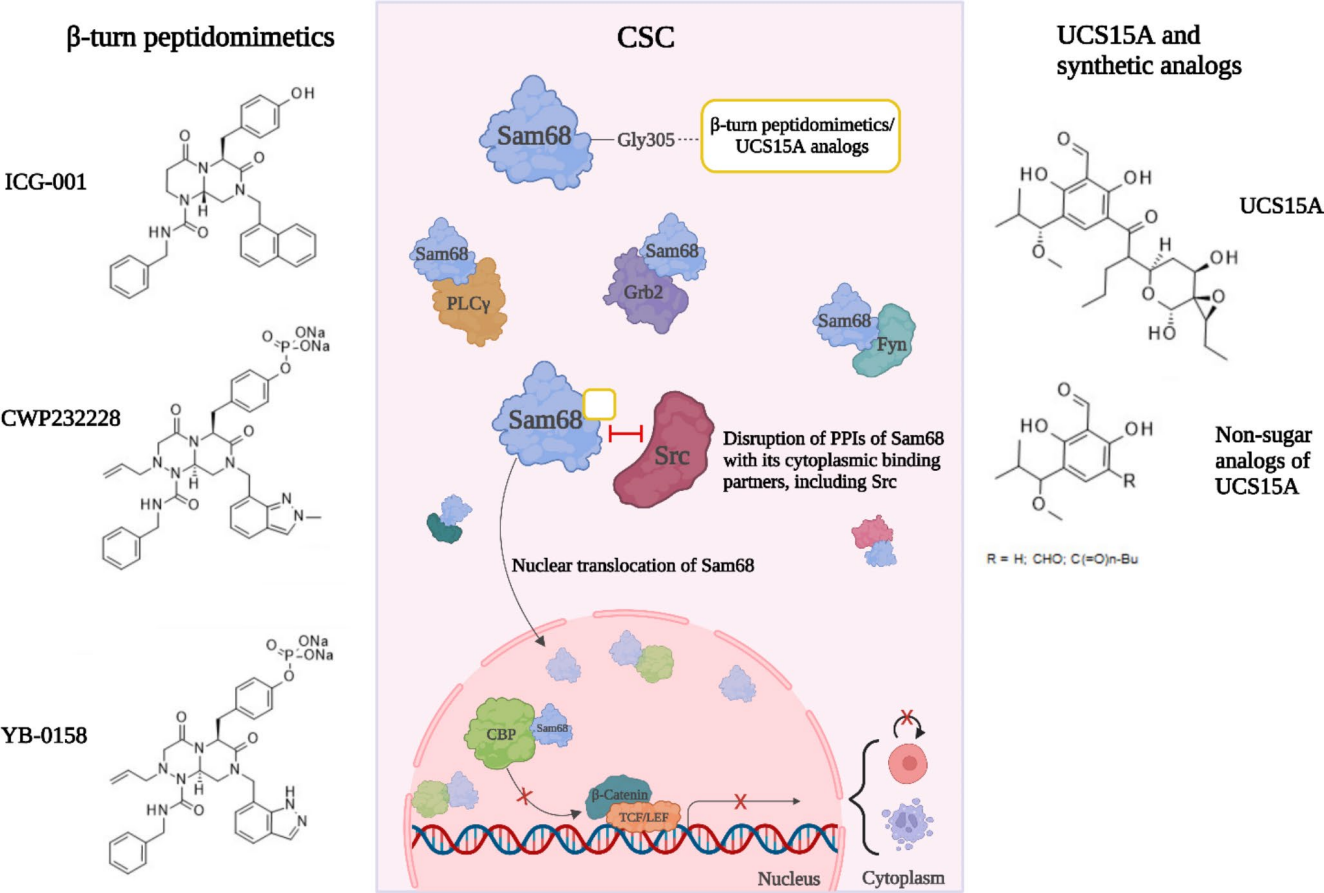
Representation of two classes of Sam68-targeting molecules (reverse-turn peptidomimetics and UCS15A-like molecules) and their proposed mechanism of action. The binding of those compounds to target protein disrupts cytoplasmic protein-protein interactions of Sam68 and leads to its nuclear translocation. There, Sam68 functions as a transcriptional repressor of Wnt signaling, leading to the blockade of self-renewal and apoptosis

Strategies based on downregulating Wnt/β-catenin signaling were successful at targeting CSC in preclinical settings, and Sam68 was reported as a crucial mediator of this transduction pathway. For instance, the reverse-turn peptidomimetic molecule ICG-001 identified through a screening campaign based on a β-catenin-responsive reporter assay (Fig. 3), was shown to induce apoptosis and growth inhibition in colorectal cancer cells, but not in normal colon epithelial cells [21]. Notably, in vivo, administration of ICG-001 reduced tumor incidence and volume in the spontaneous intestinal tumorigenesis APC^Min^ model and in a human-to-mouse colorectal cancer xenograft model, respectively [21]. Additional screening efforts identified the ICG-001 analog CWP232228 as a superior inhibitor of β-catenin dependent transcriptional activity compared to its parent molecule (Patent: WO-03031448-A1) (Fig. 3) [22]. Akin to ICG-001, CWP232228 displayed selective inhibitory effects on leukemic CSC functions, with no deleterious impact observed in health hematopoietic progenitors when tested in bone marrow serial transplantation assays [20]. However, no clear mechanistic data supported the action of reverse-turn peptidomimetics on β-catenin dependent transcriptional activity in complex biological systems, other than reduced CBP association with β-catenin and canonical Wnt target loci [20, 21]. Thus, Benoit et al. observed that ICG-001 and CWP232228 effects on CSC-associated functions were dependent on Sam68 expression levels [20].

Interestingly, ICG-001 and CWP232228 both induced an increase in Sam68 nuclear localization in cancer cells, while this phenomenon was not observed in normal stem cells [20]. Such a context-specific nuclear accumulation of Sam68 was described as SUMOylation-dependent since both compounds failed to accumulate Sam68 in the nucleus of t-hESCs overexpressing a mutant of Sam68 missing a central SUMO acceptor site (K96R) [20]. Protein SUMOylation itself was found increased in neoplastic stem-like tissues compared to healthy cells in various systems, explaining why CSCs would retain Sam68 in the nucleus more efficiently compared to normal stem cells [20, 113, 114].

Protein-protein interaction studies suggested that, once in excess in the CSC nucleus, Sam68 exerts a sequestering effect on CBP by impeding its recruitment at chromatin-bound TCF/β‑catenin complexes, therefore reducing canonical Wnt target expression (Figs. 2 and 3) [20, 24]. Again, this phenomenon was not observed in normal stem cells [20]. Increased Sam68-CBP complex formation in response to reverse-turn peptidomimetics was associated with a global decrease of CBP-catalyzed lysine acetylation marks (H3K14/18ac) in t-hESCs, supporting important modulations of CSC epigenome (Figs. 2 and 3) [20, 24]. Affinity pull-down assays in human pluripotent cell lysates confirmed the physical interaction between the active form of CWP232228 and Sam68 [24]. Competition experiments using an excess of soluble ICG-001 dissociated the interaction between Sam68 and substrate-immobilized active CWP232228, suggesting Sam68 as a direct target of both reverse-turn peptidomimetics [24].

In addition to the increased magnitude of Sam68-CBP interactions due to nuclear accumulation of Sam68, reverse-turn peptidomimetics also impact the alternative splicing of the Bcl-x transcript in t-hESCs and human AML cells [20]. Specifically, treatments with CWP232228 yielded higher levels of the Bcl-x short (Bcl-x(s)) transcript encoding a pro-apoptotic Bcl-2 homologue [20]. This switch in the abundance of Bcl-x(s) transcript reflects early observations by Paronetto and colleagues [48], where up-regulation of nuclear Sam68 shifts the splicing equilibrium towards the pro-apoptotic Bcl-x(s). Still, the specific impact of reverse-turn peptidomimetics on Sam68 functions, such as transcriptional co-regulation and alternative splicing of various transcripts, as reviewed above, warrant further investigations.

Interestingly, the nuclear accumulation of Sam68 driven by reverse-turn peptidomimetics resulted in enhanced expression of p65-dependent NF-kB target genes in HT29 cells treated with either ICG-001, CWP232228, or YB-0158 [24]. The latter is another reverse-turn peptidomimetic with enhanced CSC-targeting capacities in vivo (Tables 1**and** Fig. 3) [24]. Despite supporting previous reports on the essentiality of nuclear Sam68 in NF-kB activation, these results are accompanied by substantial induction of apoptosis which differs from the pro-survival and radioprotective role of Sam68 proposed by Fu et al. in colorectal tumors [98]. However, it is noteworthy that some NF-kB target genes encoding components of the death receptor pathway (e.g., Fas, DR4/5, TRAIL) exert pro-apoptotic functions in different cancers [115]. Accordingly, the expression of death receptor DR5 (TNFRSF10B) was significantly upregulated in HT29 cells treated with ICG-001 and YB-0158 reverse-turn peptidomimetic [24]. Moreover, nuclear p65 itself can directly induce apoptosis in cancer cells [116]. Thus, it is possible that reverse-turn peptidomimetic compounds act as context-specific regulators of Sam68-dependent NF-kB target genes in CSC.

Enhanced Sam68 expression in CSC-like cells correlates with high capacities to produce polymers of ADP-ribose (PAR) upon genotoxic stress [80]. This results from a direct interaction between Sam68 and PARP1 essential to early nuclear events of NF-kB activation following DNA-damaging insults in breast and colorectal cancer [72, 98]. Considering that reverse-turn peptidomimetic treatments significantly increase Sam68 availability to interact with PARP1, it is possible that small molecules like CWP232228 and YB-0158 can foster NF-kB-dependent pro-survival signaling and confer resistance to standard genotoxic treatments. Thus, it would be interesting to combine reverse-turn peptidomimetic treatments with PARP inhibitors to evaluate the potential synergistic effect of Wnt/β‑catenin and NF-kB inhibition in CSC functions and long-term response to camptothecin, 5-fluorouracil, oxaliplatin or radiation therapy.

### Targeting Sam68 with natural product derivatives

Previous studies highlighted the possibility of targeting Sam68 functions with small molecules such as UCS15A (Table 1), which is a bioactive compound extracted from *streptomyces* initially identified as a disruptor of Src tyrosine kinase protein-protein interactions (Fig. 3) [117]. UCS15A was reported to interfere with the interactions between the Src SH3 domain and P4/P5 proline-rich motifs present in the Sam68 [58]. Further investigation revealed that UCS15A and its synthetic analogs can also disrupt Sam68-Fyn and Sam68-Grb2 interactions, supporting that this class of compounds directly binds to Sam68 P4 and P5 proline-rich motifs (Fig. 3) [118]. Sam68 was eventually confirmed as a direct target of UCS15A in HCT116 cells via drug-induced conformational change assays performed on whole cell lysates [119].

Recently, Masibag et al. used the interaction model of UCS15A with Sam68 P3 to P5 proline-rich domains (Sam68 275–374) as a template for an *in silico* screening of a virtual library of known and hypothetical reverse-turn peptidomimetic structures [24]. Such a screen aimed to identify small molecules with enhanced binding affinity for Sam68 and display optimal selective toxicity in colorectal cancer models versus normal cells. This study identified the reverse-turn peptidomimetic YB-0158 as a compound capable of disrupting Src-Sam68 interaction and causing Sam68 nuclear accumulation in colorectal cancer cells [24]. *In silico* modeling and mutagenesis experiments highlighted the residue G305 as critical to the interaction between YB-0158 and Sam68 (Fig. 3) [24]. As for CWP232228 and ICG-001, YB-0158 downregulated Wnt target genes by sequestering CBP from the chromatin [24]. YB-0158 was also found more potent than CWP232228 at suppressing colorectal CSC activity in vivo, by using gold-standard serial tumor transplantation assays [24]. In these in vivo studies, YB-0158 demonstrated no deleterious effects on healthy gut architecture and no changes in animal behavioral or clinical indicators, supporting the cancer-selective aspect of Sam68 modulation by reverse-turn peptidomimetics.

Taken together, Sam68 represents a druggable target with the potential to selectively inhibit CSC functions whilst having minimal impact on healthy progenitor cells. Small molecules binding Sam68 and modifying its functions in cancer are already considered potential therapeutics to block CSC activity. Additional work could uncover safer and more potent candidates to be tested in pre-clinical and clinical settings.

## Future perspectives on Sam68-targeting drug discovery

Reverse-turn peptidomimetics show much promise in Sam68-focused approaches to disrupt neoplastic self-renewal, pluripotency networks, and EMT-related functions in CSC populations [24]. Further development of reverse-turn peptidomimetics with optimal affinity to Sam68 and enhanced drug-like properties could lead to novel, clinically applicable agents eliminating CSCs. The compound CWP232291 (or CWP-291) was previously reported as a Sam68-targeting peptidomimetic although its structure is yet undisclosed [120, 121]. CWP-291 reached phase-1 and 2 clinical trials for refractory multiple myeloma and AML, respectively (NTC02426723, NCT03055286) [122]. The authors reporting phase-1 data on CWP-291 administered to AML patients suggested further study of the impacts of the drug on CSC activity during subsequent phases of clinical development, considering parallels with other Sam68-targeting peptidomimetics like CWP232228 [122]. Moreover, in vivo studies in a murine spontaneous intestinal tumorigenesis model showed that CWP-291 treatments reduced tumor incidence and invasiveness [123]. This phenomenon was accompanied by reduced tumor-initiating activity and decreased percentages of cells displaying CSC markers (Lgr5, CD133) upon in vivo and ex vivo treatments with CWP-291 [123]. It will be interesting to find out whether CWP-291 can selectively suppress CSC activity in human patients while sparing normal stem cell functions in healthy tissues, and how much structural similarities this molecule shares with Sam68-targeting peptidomimetics like CWP232228 and YB-0158.

Other fascinating avenues for the discovery of new molecules disrupting Sam68 functions in CSCs reside in the current knowledge on UCS15A and its structural analogs isolated from natural extracts. Streptomyces extracts luminacins D and migracins A and B were shown to exert anticancer effects in multiple types of cancer cells through the induction of apoptosis and autophagy, or by the inhibition of cell migration, proliferation, EMT, and tumor-initiating capacity [124–126]. Specifically, in vivo injections of a synthetic analog of luminacin D reduced primary tumor burden and restricted metastasis in a xenograft model of ovarian cancer [124]. While different mechanisms of action were suggested for these UCS15A-related structural analogs, none of these studies considered a potential implication of SH3-dependent Sam68-Src interactions in the response to these compounds in cancer cells. As for UCS15A itself, the impact of the above-mentioned natural extracts and their synthetic derivatives on Sam68 subcellular distribution, CBP recruitment to canonical Wnt target genes, and CSC functions would warrant further investigation. However, it is understood that the synthesis and larger-scale production of certain UCS15A-like compounds may represent a challenge for future in vivo and clinical investigations.

It is important to mention that UCS15A is a unique non-canonical inhibitor of Src, acting via disruption of SH3 domain-mediated interactions but without directly blocking Src tyrosine kinase activity [117]. Unlike UCS15A and reverse-turn peptidomimetics, the treatment of colorectal cancer cells HCT116 with ATP-competitive inhibitor of Src family kinases PP2 had no impact on Src-Sam68 complex formation [58]. However, Paronetto et al. showed that treating prostate cancer cells with PP2 inhibitor affected cyclin D1 splicing by increasing cyclin D1b/cyclin D1a transcript ratios, which suggest that reduced tyrosine phosphorylation of Sam68 favors the expression of the pro-oncogenic isoform of cyclin D [88]. Altogether, these findings highlight the importance of disrupting Sam68 protein-protein interaction to achieve anti-tumor effects and underscore the need of further investigation on other classes of molecules potentially altering the functions of Sam68 in neoplastic stemness.

Importantly, as Sam68 is a multifunctional protein involved in many homeostatic processes, and potential pleiotropic influence of targeting Sam68 needs to be well-characterized. Further drug discovery attempts should focus on the disruption of Sam68 protein-protein interactions influencing its scaffolding functions in signaling pathways, as well as blocking the interaction of Sam68 with specific target RNAs in cancer.

## Concluding remarks

Among the multiple roles of Sam68 as an RNA-binding and adaptor protein in homeostasis and tumorigenesis, the dependence of self-renewal and tumor-initiating functions on Sam68 localization in CSCs particularly warrants attention. Sam68 is overexpressed in various cancers and higher levels of Sam68 are correlated with neoplastic stem-like properties. Examples of Sam68-targeted therapies developed to date include reverse-turn peptidomimetic compounds, which efficiently impair self-renewal capacity and alter critical hallmarks of human colorectal CSCs through Sam68-mediated disruption of CBP-β-catenin interaction. Blockade of Sam68 interactions with Src family proteins using other protein-protein disruptors, such as UCS15A, or CBP-selective PROTAC degraders may lead to a similar CSC-anticancer activity. Therefore, future investigations on targeting the Sam68 axis in CSC should uncover novel strategies to develop high-precision tools to suppress cancer stemness in the clinic.

## Data Availability

Not Applicable. This article does not involve original data.
